# Not a Produce Broker

**Published:** 1919-12-13

**Authors:** J. P. Lucas

**Affiliations:** Dunster House, Mincing Lane, E.C.


### THE EDITOR'S LETTER-BOX.

[Correspondence on all subjects is invited, but we cannot in any way be responsible for the opinions expressed by our correspondents, who must give their name and address as a guarantee of good faith, but not necessarily for publication. Correspondents are reminded that brevity of style and conciseness of statement greatly facilitate early insertion.]

### Not a Produce Broker.

*To the Editor of* THE HOSPITAL.

SIR,—In your comment last week on my letter to Dr. Addison relative to the Nurses' Registration Bill, you erroneously describe me as "Colonial Produce Broker, of Dunster House, Mincing Lane, E.C.," whereas I am not engaged in any business, and retired from the firm bearing my name some time since, although long previously I was interested in matters connected with nursing.

As this statement is calculated to detract from any useful purpose of my letter, in the theory which almost seems implied—that a "Colonial Produce Broker" should restrict his activities to his "produce," and leave other matters alone—I am entitled to make my explanation.

As a matter of fact, I have for nearly *thirty years* been almost continuously in contact with every grade of nursing, from matrons to probationers, and at the present time have both relatives and numerous friends engaged in the profession, not only in London, but in Scotland, and also in Ireland.

I think, therefore, I may claim to more knowledge of this subject of nurses' hours than would usually be credited to a "Colonial Produce Broker," and my letter to Dr. Addison is merely following up the suggestions I advanced a year ago through the hospitality of your columns (December 21), and in continuation of much work I have since put in on behalf of overworked nurses.

I regret that you should consider I fall into "several errors" in the course of my letter; but I must confess to being puzzled as to where they are.

For the reasons I have given, and as I am not disposed to abandon my right to an endeavour to help forward the cause of hospital nurses, I will ask you to be good enough in your next issue to remove the impression you create by reference to my previous business.

I am convinced, by a knowledge of facts around me, that reforms for nurses are urgently necessary, but there is no man who can say that I have ever belittled the work of any hospital in my life; nor, so far as I am aware, am I at war with anyone.

Yours faithfully,
J. P. LUCAS.

Dunster House, Mincing Lane, E.C.
*December* 6, 1919.

